# Self-regulation training for people with knee osteoarthritis: a protocol for a feasibility randomised control trial (MiNT trial)

**DOI:** 10.3389/fpain.2023.1271839

**Published:** 2024-01-08

**Authors:** Ramakrishnan Mani, Divya Bharatkumar Adhia, Sharon Awatere, Andrew Robert Gray, Jerin Mathew, Luke Charles Wilson, Amanda Still, David Jackson, Ben Hudson, Fadel Zeidan, Roger Fillingim, Dirk De Ridder

**Affiliations:** ^1^ Centre for Health, Activity and Rehabilitation Research, School of Physiotherapy, University of Otago, Dunedin, New Zealand; ^2^ Department of Surgical Sciences, Otago Medical School, University of Otago, Dunedin, New Zealand; ^3^ The Health Boutique, Napier, New Zealand; ^4^ Biostatistics Centre, University of Otago, Dunedin, New Zealand; ^5^ Department of Anatomy, School of Biomedical Sciences, University of Otago, Dunedin, New Zealand; ^6^ Otago Medical School, University of Otago, Dunedin, New Zealand; ^7^ Department of General Practice, University of Otago, Christchurch, New Zealand; ^8^ Department of Anesthesiology, School of Medicine, University of California, San Diego, CA, United States; ^9^ Pain Research and Intervention Center of Excellence, Clinical and Translational Science Institute, College of Dentistry, University of Florida, Gainesville, FL, United States

**Keywords:** chronic pain, musculoskeletal pain, mindfulness meditation (MM), mindfulness—pain intervention, neurofeedback (NF), self-regulation training, knee osteoarthritis, feasibility and acceptability

## Abstract

**Introduction:**

Knee osteoarthritis (OA) is a chronic secondary musculoskeletal pain condition resulting in disability, reduced quality of life, and high societal costs. Pain associated with knee OA is linked to increased sensitivity in sensory, cognitive, and emotional areas of the brain. Self-regulation training targeting brain functioning related to pain experience could reduce pain and its associated disability. Self-regulatory treatments such as mindfulness meditation (MM) and electroencephalography neurofeedback (EEG-NF) training improve clinical outcomes in people with knee OA. A feasibility clinical trial can address factors that could inform the design of the full trial investigating the effectiveness of self-regulation training programmes in people with knee OA. This clinical trial will evaluate the feasibility, safety, acceptability, experience and perceptions of the self-regulatory training programmes.

**Methods:**

The proposed feasibility trial is based on a double-blind (outcome assessor and investigators), three-arm (MM usual care, EEG-NF + usual care and usual care control group) randomised controlled parallel clinical trial. Participants with knee OA will be recruited from the community and healthcare practices. A research assistant (RA) will administer both interventions (20-min sessions, four sessions each week, and 12 sessions over three successive weeks). Feasibility measures (participant recruitment rate, adherence to interventions, retention rate), safety, and acceptability of interventions will be recorded. An RA blinded to the group allocation will record secondary outcomes at baseline, immediately post-intervention (4th week), and 3 months post-intervention. The quantitative outcome measures will be descriptively summarised. The qualitative interviews will evaluate the participants' experiences and perceptions regarding various aspects of the trial, which includes identifying the barriers and facilitators in participating in the trial, evaluating their opinions on the research procedures, such as their preferences for the study site, and determining the level of acceptability of the interventions as potential clinical treatments for managing knee OA. Māori participant perceptions of how assessment and training practices could be acceptable to a Māori worldview will be explored. The interviews will be audio-recorded and analysed thematically.

**Discussion:**

This trial will provide evidence on the feasibility, safety, and acceptability of the MM and EEG-NF training in people with knee OA, thus informing the design of a full randomised clinical control trial.

## Introduction

Knee osteoarthritis (OA) is a chronic secondary musculoskeletal pain condition resulting in disability, reduced quality of life, and high societal costs (1, 2). Knee OA was among the top-ranked conditions in terms of the number of years lived with disability (3). Chronic pain in knee OA is often attributed to structural changes in joint structures. However, there is an apparent discordance between the nature and intensity of the pain and the degree of structural changes in the knee joints (4, 5). Pain associated with knee OA is linked to increased sensitivity in sensory, cognitive, and emotional cortices of the brain and psychological contributions (6–17). Self-regulation training targeting normalising brain functioning related to pain experience could improve clinical outcomes (18–24).

Mindfulness meditation training (MMT), a form of self-regulation training, ‘*involves focussed attention to the changing sensations of the body (usually the breath) and non-reactive monitoring of arising sensory events*’ (19). Several mechanisms that mediate pain relief following MMT have been identified (25). Neuroimaging studies confirm that MMT can modulate activity in the cortical areas processing sensory, cognitive, and emotional components of the pain experience. MMT improves mood, increases autonomic modulation, and engages non-opioid mechanisms that could mediate pain outcomes (26–38).

Electroencephalography neurofeedback (EEG-NF) is a biofeedback training designed to improve health outcomes. The EEG-NF training involves assisting people to learn to self-regulate the activity of specific brain areas that are involved in key functions related to pain perception and disease. In the EEG-NF training, a real-time feedback (reward) is provided when the participant's brain activity reaches the set threshold frequency level of the targeted cortical electrical activity (39–48). Evidence demonstrates a clinically significant reduction in pain following the EEG-NF training (49–55). NF also improves anxiety, emotional regulation, brain activity, and autonomic modulation, potentially mediating mechanisms of pain reduction (56–63). Pilot EEG-NF training targeting infraslow frequency bands demonstrated encouraging patterns for pain outcomes in people with chronic musculoskeletal pain (64, 65).

Both MMT and NF are stand-alone, promising self-regulatory interventions demonstrated to improve pain outcomes. However, evidence of the effectiveness of these two self-regulation training methods is lacking in people with knee OA, a common chronic secondary musculoskeletal pain condition. Although both interventions appear to rely on self-regulatory principles, the physiological and psychological mechanisms through which these two interventions improve pain outcomes may be different (63–69). Therefore, identifying the mechanisms for how these self-regulatory interventions improve outcomes can assist in further optimising protocols to enhance outcomes. Thus, the primary aim of the full clinical trial will be to assess the clinical effectiveness and cost-effectiveness of MMT + usual care and EEG-NF training + usual care against usual care for improving pain and functional outcomes in individuals with knee OA. The secondary aim of the full randomised clinical trial will be to identify the psychological and physiological mechanisms by which training reduces pain and improves function.

However, prior to conducting this full RCT, we propose conducting a feasibility trial to address the factors that could influence the design of the full clinical trial (64). The feasibility issues/questions that require understanding prior to conducting a full-powered RCT are as follows: Are mindfulness meditation and neurofeedback training feasible, safe, and acceptable interventions for people with knee OA? What are the participant recruitment, enrolment, adherence, and dropout rates? What are the adverse side effects of these two self-regulation-based training programmes? What are the participant's experiences of the treatment and research procedures, particularly Māori perceptions of how assessment and training practices in the trial are acceptable to cultural perspectives and values, including Te Ao Māori (a Māori worldview)? What modifications are required for the full trial? What sample size is required to conduct an adequately powered trial?

Therefore, the objectives of this feasibility randomised clinical trial are as follows:

• Objective 1: To determine the feasibility (i.e., rates of participant recruitment, enrolment, training compliance, retention/dropouts) of conducting the full randomised clinical trial.
• Objective 2: To examine the safety of the administered interventions.
• Objective 3: To explore the participants' perceptions regarding the study procedures, including the acceptability of interventions.
• Objective 4: To explore Māori perceptions of how assessment and training practices in the trial are acceptable to cultural perspectives and values, including Te Ao Māori (a Māori worldview).
• Objective 5: To derive the central tendency and variability of the clinical outcome measures to inform the sample size of a full clinical trial.

## Research design and methods

This protocol was reported in accordance with the CONSORT statement on pilot/feasibility studies (69). This trial is prospectively registered in the Australian New Zealand Clinical Trials Registry (ANZCTR 12621001741875). The trial has been documented following the recommendations outlined in the Interventional Trials (SPIRIT) statement and the template for intervention description and replication (TIDieR) checklist (Table 1) (70).

**Table 1:** Description of the EEG-NF and MMT, based on the template for intervention description and replication.

Item number and item	Description
1. **Brief name** Provide the name or a phrase that describes the intervention.	Mindfulness meditation training (MMT) Electroencephalography neurofeedback (EEG-NF) training
2. **Why** Describe any rationale, theory, or goal of the elements essential to the intervention.	Infraslow frequency (<0.1 Hz) is a brain electrical rhythm with maximal spectral power in frequencies below 0.1 Hz. The phase of ISF is correlated with amplitudes of higher frequency bands. Altering ISF may normalise electrical activities across resting-state brain networks that are altered in people with chronic pain (17). The region of interest for EEG-NF training is the pregenual anterior cingulate cortex (pgACC). pgACC is a cortical area processing emotional information and is functionally connected to brainstem centres responsible for endogenous inhibitory function. The EEG-NF training programme is designed to up-train the ISF at the pregenual anterior cingulate cortex (pgACC) to promote greater modulation of sensory inputs. A brief MMT regimen involves participants focussing on the sensation of each breath occurring at the tip of the nose. MMT regimen will influence psychological processes, including anxiety, cognition and attention and promote pain relief via non-opioid mechanisms.
3. **What** Materials: Describe any physical or informational materials used in the intervention, including those provided to participants or used in intervention delivery or training of intervention providers. Provide information on where the materials can be accessed (e.g., online appendix, URL).	Each participant will wear an EEG cap with sensors fixed to the scalp and connected to a 21-channel DC-coupled amplifier (BrainMaster Technologies, Inc.). ISF sLORETA software (Neurofeedback Services of New York) will be used for ISF-NF training.
4. **Procedures:** Describe each of the procedures, activities, and/or processes used in the intervention, including any enabling or support activities.	Participants will be seated. Participants will be instructed to close their eyes, relax, and listen to the sound feedback. The participants will be instructed to minimise eye blinks, eye/head/neck movements, swallowing manoeuvres, and teeth clenching. The software programme will play a distinct tone when the brain activity of participants reaches the set threshold of infraslow magnitude at the pgACC. The RA-1 will be trained remotely by the Centre for Mindfulness (UCSD) to provide an MMT regimen in the trial. MMT involves instructing participants to focus on the sensation of each breath occurring at the tip of the nose. As a progression, the participants will be instructed to broaden their focus to the ‘full flow of the breath’, including bodily sensations. The participants will be instructed to acknowledge thoughts, feelings, and emotions as they arise without judgment and ‘simply return their attention back to the breath sensations’.
5. **Who provided** For each category of intervention provider (e.g., psychologist, nursing assistant), describe their expertise, background, and any specific training given.	A research assistant trained in delivering both interventions will administer the training.
6. **How** Describe the modes of delivery (e.g., face-to-face or by some other mechanism, such as internet or telephone) of the intervention and whether it was provided individually or in a group.	All participants will receive individual face-to-face training sessions.
7. **Where** Describe the type(s) of location(s) where the intervention occurred, including any necessary infrastructure or relevant features.	Interventions will be delivered in the School of Physiotherapy laboratory, University of Otago, Dunedin, New Zealand.
8. **When and how much** Describe the number of times the intervention was delivered and over what period of time, including the number of sessions, their schedule, and their duration, intensity, or dose.	The participants will undergo training for 12 sessions, four times a week, for three consecutive weeks. Each training session will last for a 30 min duration.
9. **Tailoring** If the intervention was planned to be personalised, titrated or adapted, then describe what, why, when, and how.	The BrainMaster training programme will provide feedback (reward) according to the real-time brain states of the study participants.
10. **Modifications** If the intervention was modified during the course of the study, describe the changes (what, why, when, and how).	Not applicable. This is a protocol for a feasibility trial.
11. **How well** Planned: If intervention adherence or fidelity was assessed, describe how and by whom, and if any strategies were used to maintain or improve fidelity, describe them.	A research assistant providing training will record the number of training sessions attended by participants (i.e., adherence rates). Adherence rates will be summarised at the end of training. An investigator will monitor the fidelity of the intervention.
12. **Actual:** If intervention adherence or fidelity was assessed, describe the extent to which the intervention was delivered as planned.	Not applicable. This is a protocol for a feasibility trial.

### Study design

This feasibility study (71, 72) will be a double-blind (outcome assessor and all investigators), three-arm randomised control parallel trial with participants having an equal probability of being assigned to each arm. The study outcome measures will be collected at baseline, immediately post-intervention (4th week), and 3 months post-intervention.

### Ethical approval

Ethical approval was obtained from the NZ Health & Disability Ethics Committee. The Ngāi Tahu Research Consultation was obtained. The School of Physiotherapy health and safety committee will act as an internal Data and Safety Monitoring Board.

### Establishing whakawhanaungatanga (relationships) with Māori

Osteoarthritis is an increasing health condition among the Māori population. Improving the OA treatment will help in reducing health inequalities between Māori and non-Māori (17, 73–80). Combining our consultation with the Ngāi Tahu Research Committee, Māori researchers, and research work conducted by Māori on chronic pain, we will implement practices acceptable to a Māori worldview, thus being responsive towards the cultural and health needs of the Māori people. The clinical trial will be guided by a Māori-centred research approach (i.e., Kaupapa Māori research) that incorporates cultural considerations and provides an interpretive framework aligned to Māori customs (tīkanga) base. A Māori researcher (SA) with expertise in issues about kaumātua living with OA will oversee the implementation of tīkanga principles and administer culturally appropriate strategies to recruit Māori participants. We will adhere to Māori rituals of engagement, such as pōwhiri and hui, on Marae and wharenui (meeting house in Marae). This will involve presenting the background and procedures of the study, as well as the findings from previous work on lived experiences of pain among Māori. In addition, we will discuss culturally appropriate pain assessment tools for Māori with OA, as well as involve their family and whānau in the community. Hui will be carried out face-to-face (kanohi-ki-te-kanohi) to enable the community to see who the researchers are and include whanaungatanga (strengthening connections). Engaging participants with respectful behaviour closely related to Māori customs, such as ‘aroha’ and ‘manākitanga’. The priority will be getting the community talking about interventions for reducing pain, improving function, and strengthening referral networks of Māori living with OA into the research. A Māori researcher will conduct interviews with Māori participants. In hui and interviews, as well as assessment and intervention sessions, the importance of whānau support means that Māori participants can bring families and friends for the assessment and treatment sessions. Furthermore, koha (such as food, drink, and a voucher) will be offered as a mark of appreciation at each interview. In prioritising mana (respect) for the participants, Māori culture considers the head sacred (‘he tapu te upoko’). Therefore, permission must be obtained at every EEG-NF session before touching the participant's head. Finally, the research outcomes will be fed back to the community to offer nurturing guidance (awhi) and community relationship building. In this way, the research process and outcomes form lasting relationships beneficial to both the research and the community.

## Study eligibility criteria

The inclusion criteria of the study are as follows: Adults (45–85 years) will be eligible to participate if they meet the clinical criteria for the diagnosis of knee OA according to the guidelines established by the American College of Rheumatology (ACR) and with knee pain that persisted for longer than 3 months, (81). The exclusion criteria of the study are as follows: Recent soft tissue injuries of the knee (e.g., ligaments, muscles, meniscus, tendon) in the last 3 months, infective/inflammatory arthritis, scheduled for joint replacement surgery within the next 4 months, joint injections (hyaluronic acid) in the last 6 months, joint injections (steroids) in the last 3 months, brain injury or diseases (e.g., stroke, multiple sclerosis, Parkinson's disease), spinal cord injury or diseases, nerve injuries or neuropathy in the legs, migraine or recurrent headaches, cognitive illness (dementia)—any difficulty thinking, reasoning, or remembering, major psychiatric illnesses and former neurosurgical procedures of the brain, and recent/current pregnancy.

## Study location and settings

### Recruitment strategy

We will recruit participants from the wider Dunedin community. Community newspapers [The Star (a free newspaper published weekly) and the Otago Daily Times (a paid newspaper published daily)] will periodically publish study advertisements, reaching out to the urban area of Dunedin city, its suburbs, and other nearby villages. Sponsored Facebook adverts will also be implemented. Targeted advertisements will be published in community organisations, including arthritis advocacy groups and community-based exercise classes. Patients attending primary healthcare practices based in Dunedin will also be invited to participate.

The participants in the experimental groups will get a reimbursement of $200 in the form of supermarket/travel vouchers as a compensation for their time dedicated to this study. It is anticipated that each participant will contribute approximately 20 h to the study, including three 2-h assessments [baseline, post-intervention (4th week), and 3 months post-intervention], 12 1-h training sessions, and 1 h of a qualitative interview.

The participants in the usual care group will get a reimbursement of $80 in the form of supermarket/travel vouchers as a compensation for their time dedicated to this study. It is anticipated that each participant will contribute approximately 8 h to the study, which includes three 2-h assessments and 1 h of a qualitative interview.

### Māori sampling

The purposeful sampling strategy will be conducted with people identifying as being of Māori ancestry and ethnicity that meet our sampling criteria. Key informants will initially be identified through the Māori researcher and research teams' whānaungatanga (networks and relationships) and Māori healthcare providers (73). The snowball sampling method will involve asking the initial respondents to suggest others, precisely who they know in the target group and whom to contact and invite to take part in the research. A Māori-specific participant information sheet will clearly outline the purpose of the study and emphasise that the information provided by Māori participants, their family, and their whānau could contribute to the acceptability of the study as a potential clinical treatment for the management of knee OA from a Māori perspective. A Māori investigator will train the research staff on Tikanga Māori to create a safe and respectful atmosphere for all participants during the trial.

## Screening and enrolment

Volunteers who expressed interest in participating in the study will undergo initial screening via a digital survey or phone. The participants will undergo screening for cognitive involvement at the beginning of the baseline assessment, and if found eligible, they continue to complete the baseline assessment. The following procedures will be conducted at the baseline assessment.

### Baseline assessment

Written informed consent will be obtained, and the participants will be subjected to a baseline assessment to complete the questionnaires (age, sex, ethnicity, education, income, employment status, OA duration, pain severity, interference and quality, co-morbidity, psychological states, medication use, and other treatments for OA pain-management activities) (82–84) and undergo anthropometric measurements.

### Allocation

After the baseline assessment, the participants will be allocated into one of the three study groups with equal probabilities. Blocks of unequal length, with the measurements and the possibilities of these lengths unknown to other investigators, will be used to promote allocation concealment. The senior biostatistician, using non-informative group allocation codes, will generate the allocation sequence using a standard computer software.

### Allocation concealment

The allocation of groups will be concealed using opaque envelopes to conceal the sequence until interventions are assigned. The senior biostatistician will prepare the envelopes and provide them to the assistant research fellow (ARF). The ARF will give the opaque sealed envelope to the participants following the baseline assessment. The participants open the envelope to reveal the group they have been allocated to after the baseline assessment. The participants inform the ARF about the group, and the ARF administers one of the active interventions to the participant. If a participant is identified to be allocated to the usual care control group, they would be advised to continue managing their health condition as usual and attend the follow-up assessment sessions.

### Blinding

The outcome assessor and all investigators will be blind to the group allocation. The participants and the intervention provider will not be blinded to the intervention because the content will be evident. The biostatistician will be blinded to the study groups until all planned analyses are completed, thus minimising bias during data analysis. All the participants will be requested to keep their treatment allocation group private from the outcome assessor during the duration of the study. This information is detailed and indicated to the participants within the study information sheet.

### Interventions

Following the baseline assessment and group allocation, the participants will receive one of the active interventions or the usual care control group. The number of sessions and duration for both interventions will be identical. A trained research assistant will administer both active interventions. A research assistant will be trained to administer the study interventions. Both interventions will consist of 12 20-min sessions which will be administered in four consecutive sessions/week for three successive weeks. The interventions described in this study adhered to the Template for Intervention Description and Replication guide.

### MMT

An MMT regimen (85) will be used. An RA will be trained remotely by the Centre for Mindfulness, University of California San Diego, to conduct the intervention. During each session, the participants will be instructed to focus on the sensation of each breath occurring at the tip of the nose. On progression, the participants will be instructed to broaden their focus to the ‘full flow of the breath’, including bodily sensations. We will instruct the participants to acknowledge thoughts, feelings, and emotions as they arise without judgment and ‘simply return their attention back to the breath sensations’.

### EEG-NF training

**Figure 1:**
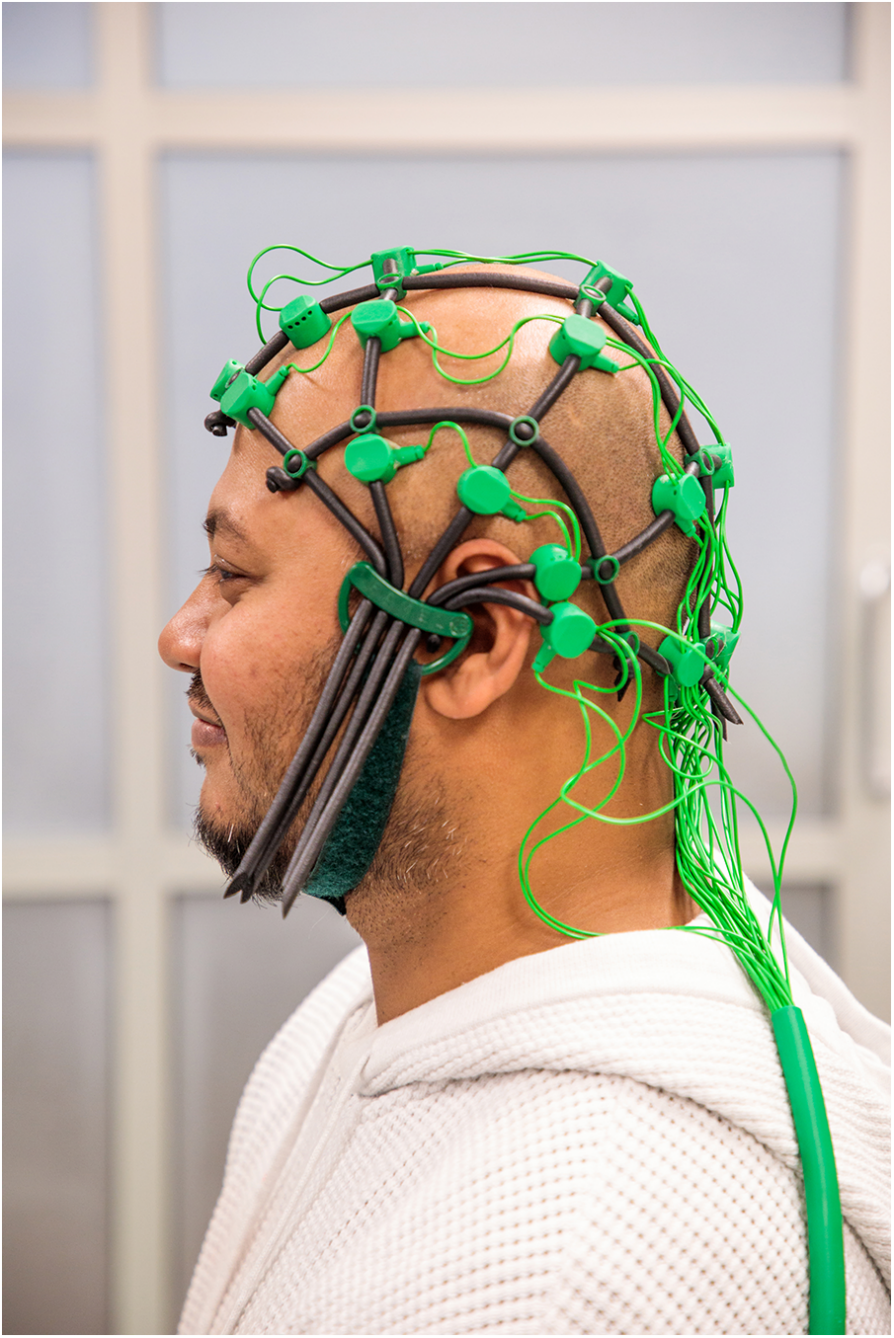
A model wearing an EEG Combi Cap.

An RA will be trained to administer the EEG-NF training using the BrainMaster Inc. system (86). Comby EEG Caps with sensors (Ag/AgCl) are used to record EEG signals (Figure 1). The participants will be instructed to minimise eye blinks, eye/head/neck movements, swallowing manoeuvres, and teeth clenching. The software programme will play a distinct tone when the brain activity of the participants reaches the set threshold of infraslow (0.0–0.1 Hz) frequency band at the pregenual anterior cingulate cortex (pgACC). The participants will be seated and instructed to close their eyes, relax, and listen to the sound feedback. The reward threshold will be adjusted in real time between 60%–80%, which means that the sound feedback will be delivered 60%–80% of the time.

We will use the EEG- NF programme administered in our pilot studies in people with chronic musculoskeletal pain, including knee OA (64, 65). Infraslow frequency (<0.1 Hz) is a brain electrical rhythm with maximal spectral power in frequencies below 0.1 Hz. Influencing the ISF may normalise electrical activities across resting-state brain networks that are altered in people with chronic pain (87–90). The pgACC is the chosen region of interest (ROI) for the training due to its connectivity with the periaqueductal grey matter (PAG), which promotes endogenous pain inhibition (17, 90, 91). Up-training ISF at the pgACC improved the pain outcomes in people with chronic pain (65). Therefore, the EEG-NF training programme was designed to up-train the ISF at the pgACC to improve pain outcomes through promoting greater sensory modulation, altering attention and cognitive controllability of pain.

The participants in the control group who are receiving usual care will continue to receive treatments as usual. However, they will refrain from practising meditation during the study period. Following the trial completion, they will be offered to receive one of the trainings.

### Outcome measures

The assistant research fellow will collect primary outcome measures throughout the study period. All secondary outcome measures will be collected at baseline, immediately post-intervention (4th week), and 3 months post-intervention by a research assistant (RA-2) blinded to group allocation. The RA will be adequately trained to collect quantitative sensory testing procedures, electrocardiogram, and electroencephalography recordings (Table 2).

**Table 2:** Secondary outcome measures.

Outcome domain and constructs (96–98)	Brief description of measurement tools	Measurement time points
Pain		
Pain severity	The brief pain inventory—short form (pain subscale) Pain severity on a 10-point scale (0 = no pain and 10 = worst pain possible) in the past 24 h, 1 week, and 4 weeks.	T_B,_ T_1m,_ T_3m_
Pain unpleasantness	In the last 2 weeks, how unpleasant has your knee pain been? ‘Not at all’, ‘slightly’, ‘moderately’, ‘very much’, and ‘extremely’.	T_B,_ T_1m,_ T_3m_
Pain bothersomeness	In the last 2 weeks, how bothersome has your knee pain been? ‘Not at all’, ‘slightly’, ‘moderately’, ‘very much’, and ‘extremely’.	T_B,_ T_1m,_ T_3m_
Intermittent and constant pain	The measure of intermittent and constant osteoarthritis pain (11 items). Each item is rated on a 5-point ordinal response scale (Not at all/No constant or intermittent knee pain, Mildly, Moderately, Severely, Extremely).	T_B,_ T_1m,_ T_3m_
Pain quality	PainDETECT questionnaire (12 items). Each symptom is rated on a 5-point ordinal response scale (1 = never to 5 = very strongly). Score classification: ≤12—nociceptive pain, 13–18—possible neuropathic pain, ≥19—likely neuropathic pain.	T_B,_ T_1m,_ T_3m_
Movement evoked pain	Pain severity during a 6-min walk distance and physical performance tasks—rated pain on NPRS on a 0–100 scale (0 = no pain, 100 = the most intense pain imaginable) scale.	T_B,_ T_1m,_ T_3m_
Flare in osteoarthritis	Flare-OA (16 items) patient-reported instrument to assess the occurrence and severity of knee OA in the past 4 weeks.	T_B,_ T_1m,_ T_3m_
Physical functioning		
Pain interference	The brief pain inventory—short form (interference subscale—nine items) evaluate the impact of pain on daily activities Rated on a 10-point scale (0 = no pain and 10 = worst pain possible).	T_B,_ T_1m,_ T_3m_
Physical function	Knee injury and osteoarthritis outcome score (KOOS): measuring function in daily living (17 items), function in sport and recreation, and knee-related quality of life. Rated on a 5-point ordinal response scale, with anchors of zero (no problems) to 4 (extreme problems).	T_B,_ T_1m,_ T_3m_
Physical activity levels	International physical activity questionnaire—short form in the last 7 days	T_B,_ T_1m,_ T_3m_
Psychological functioning		
Depression, anxiety, and stress	Depression, anxiety, and stress scale (21 items) is a quantitative measure of psychological distress. Rated on scale: 0—never, 1—sometimes, 2—often, 3—almost always.	T_B,_ T_1m,_ T_3m_
State anxiety	The state-trait anxiety inventory will be used to measure state anxiety.	T_B,_ T_1m,_ T_3m_
Pain catastrophising thoughts	Pain catastrophising scale: 13 items; the degree of rumination, magnification, and helplessness when experiencing pain using the 0 (not at all) to 4 (all the time) scale. Total score: 0–52.	T_B,_ T_1m,_ T_3m_
Pain vigilance and awareness behaviour	Pain vigilance and awareness questionnaire (PVAQ): 16 items; the degree of vigilance to and awareness of pain using the 0 (never) to 5 (always) scale. Total score: 0–80.	T_B,_ T_1m,_ T_3m_
Pain self-efficacy	Pain self-efficacy questionnaire (two-item) measures confidence in one's ability to work and lead a normal life despite pain. Rated on 7-point scale: 0 = not at all confident and 6 = completely confident:	T_B,_ T_1m,_ T_3m_
Control of emotions	Emotional regulation questionnaire (10-item scale) measures the tendency to regulate their emotions in two ways: cognitive reappraisal and expressive suppression. Rated on a 7-point ordinal response scale ranging from 1 (strongly disagree), 4 (neutral), to 7 (strongly agree).	T_B,_ T_1m,_ T_3m_
Affect style	Positive and negative affect scale (20 items) Rated on a 5-point ordinal response scale, ranging from 1 = Very slightly or not at all to 5 = Extremely.	T_B,_ T_1m,_ T_3m_
Mindfulness	Five-facet mindfulness questionnaire (15 items) will be used to assess elements of mindfulness. Rated on a 5-point ordinal response scale, ranging from 1 = Never or very rarely true to 5 = Very often or always true.	T_B,_ T_1m,_ T_3m_
Coping levels	A brief pain coping scale will be used to assess coping strategies to manage pain.	T_B,_ T_1m,_ T_3m_
General health and well-being		
Quality of life and well-being	European quality of life-5D and WHO-5 well-being index.	T_B,_ T_1m,_ T_3m_
Sleep quality and disturbances	Pittsburgh sleep quality index (19 items) assesses sleep quality over a 1-month time interval. Scored 0 (no difficulty) to 3 (severe difficulty). Total score (range 0–21). Higher scores indicate worse sleep quality.	T_B,_ T_1m,_ T_3m_
The participants’ perceptions about interventions and their impact on their health condition/pain.		
Global perceived change	Perceived change in the knee pain when compared to baseline/before training (−5 = much worse, through 0 = unchanged, to +5 = completely, recovered).	T_1m, and_ T_3m_
Acceptability of interventions	Visual analogue scales (0–10) to measure overall acceptability, burden, perceived effectiveness, ethicality, culturally acceptable, research team's trustworthiness and knowledge, and the likelihood of negative side effects of training.	T_B_
Perceived treatment satisfaction and usefulness of training	Rated on a 0–10 NRS; 0—not at all satisfied to 10—highly satisfied.	T1m, and T3m
Credibility/expectancy of the intervention	The credibility/expectancy questionnaire (5 items)	T_B_
Predictors of training		
Level of engagement with the training	Rated on a 10-point ordinal items scale, where 1 = least engaged and 10 = highly engaged.	At every session
Level of motivation	Single-item; rated on a 10-point ordinal items scale, where 1 = least motivated and 10 = highly motivated.	T_B_ and every session
Cost-and-consequences		
Costs	The osteoarthritis cost-and-consequences questionnaire will be used to measure healthcare use/expenses in the past 3 months.	T_B,_ and T_3m_
Quantitative sensory testing, physical performance assessment and physiological recordings		
	Quantitative sensory testing procedures	T_B,_ T_1m,_ T_3m_
	Resting and evoked electroencephalography.	
	Heart rate, blood pressure, and respiratory rates	
	OARSI Minimum physical performance core set	
	Short physical performance battery (SPPB) standing balance tasks.	

T_B_, at baseline; T_1m_, immediately post-intervention (4th week); T_3m_, 3 months post-intervention.

## Primary outcomes

### Feasibility data

The feasibility data (71) will be collected by the assistant research fellow.

• Recruitment rate and enrolment: The percentage of the number of participants recruited from the total number of participants screened over 1 year will be calculated. We aim to recruit 20 participants in each arm of the trial over 1 year in a single centre.
• Compliance rate to training sessions: A research assistant providing training will record the number of training sessions attended by the participants (i.e., adherence rates). The adherence rates will be summarised as percentages. The reasons for non-compliance (e.g., adverse effects) will be documented. A targeted compliance rate of 80% will be considered acceptable.
• Participant retention rate (dropout rates): The percentage of the allocated participants in each group who completed the follow-up assessment will be measured. A targeted retention rate of 80% will be considered acceptable.

### Safety data

The training administrator will record symptoms using the Discontinuation-Emergent Sign and Symptom (DESS) (93) checklist. The 43-item checklist includes emotional, behavioural, cognitive, and physical symptoms, and the participants will be required to compare their current status with their status prior to the training or the previous session.

### Qualitative study

An assistant research fellow will coordinate with the investigators in conducting semi-structured in-depth interviews following the intervention to evaluate participant experiences in the trial (94, 95). As they finish the trial, the participants will be asked if they would consider this additional interview and be enrolled sequentially until numbers are met. The interview will explore experiences and perceptions regarding interventions based on the theoretical framework of acceptability. These would be recorded with the consent of the participants. The aims of this interview include exploring the participants’ experiences regarding the barriers and facilitators in participating in the trial. In addition, the interview aims to assess the acceptability of the research procedures, including the intervention, preferences for the study site, the perceived value of the study, and the acceptability of interventions as potential clinical treatments for managing knee OA. Furthermore, the interview seeks to explore the perspectives of Māori people on assessment and treatment practices that align with their cultural worldview (95). Māori is treated as an important subgroup and are selected and interviewed separately. We aim to interview five people per group with a purposeful sample of Māori participants from each group. The participants in the usual care group will also be interviewed to explore their experiences participating in the trial, including the acceptability of the assessment procedures.

## Secondary outcomes

Table 2 presents the outcome domain and constructs, together with a brief description of the measurement tools used to measure the constructs and the measurement time points (96–127). All outcomes are well-established, reliable, and validated constructs in people with persistent pain (predictors of pain and disability) and are recommended for clinical trials of chronic pain and knee osteoarthritis (96–98, 120). Each assessment session will last for a maximum of 2 h, including completing the questionnaires and undergoing testing.

### Outcome assessor and blinding

A research assistant who will be blinded to the groups will collect the secondary outcomes at baseline, immediately post-intervention, and 3 months post-intervention. The effectiveness of blinding will be measured. The self-report questionnaires will be administered using the Qualtrics data management system. The participants will complete the questionnaires in two parts: at home and in person during the assessment sessions.

Pain and physical function measures will be measured using validated questionnaires in people with knee OA (99–102). All participants will perform the minimum physical performance core set (sit-to-stand, 30-s chair stand test, and walking short distances) as recommended by the Osteoarthritis Research Society International (102). A short physical performance battery (SPPB) will be administered that includes standing balance tasks. Pain intensity during each physical performance will be recorded using a numerical 0–100 numeric rating scale (0 = no pain, 100 = the most intense pain imaginable) (102, 132): All participants will be asked to perform a 6-min walk test (6MWT) to derive the Sensitivity to Physical Activity (SPA) index. The participants will be asked to rate their discomfort before the 6MWT and every minute of walking. The SPA index will be derived by subtracting baseline pain ratings from their greatest pain ratings for each trial. The European Quality of Life-5 Dimensions scale will be used to assess health-related quality of life (104). Depression, anxiety, and stress will be recorded, as well as the occurrence of pain catastrophising thoughts, pain self-efficacy, positive and negative affect, emotion regulation, and elements of mindfulness and coping levels (106–112). Sleep quality and physical activity levels will be measured. Following the training, the participants' perceptions of change (−5 = much worse, through 0 = unchanged, to +5 = completely recovered) in their symptoms will be measured using the global rate of change scale (99) Visual analogue scales (0–10) will be used to measure the overall acceptability, burden, perceived effectiveness, ethicality, culturally acceptable, the research team's trustworthiness and knowledge, and the likelihood of negative side effects of training. The credibility/expectancy questionnaire will measure the treatment expectancy and rationale credibility of the interventions tested (113, 114). The level of engagement with the training sessions will be recorded using a 10-point ordinal items scale, where 1 = least engaged and 10 = highly engaged—level of motivation. For the EEG-NF group, an adapted version of the Current Motivation-Brain Computer Interference (QCM-BCI) questionnaire will be used to assess the level of motivation for participation in the training (114). The Osteoarthritis Cost-and-Consequences questionnaire will be used to record healthcare use/expenses during the 3-month period prior to the study and the 3-month period post-intervention (105).

### Cardiovascular measures

The resting heart rate and beat-to-beat R-R interval will be measured using a Polar V800 HR monitor and a Polar H10 chest Pro Strap for a duration of 7 min, with a resolution of 2 ms. Blood pressure and respiratory rate will be measured. Raw heart rate and R-R interval time series data will be downloaded from the Polar flow software for further processing and analysis. Kubios software will be used to derive time and frequency domains of heart rate variability (HRV). Peripheral oxygen saturation will be monitored and recorded (123, 124).

**Quantitative sensory testing** procedures will be administered following the guidelines (115–122).

An algometer (AlgoMed, Medoc, Ramat Yishai, Israel) will be used to measure three trials of pressure pain threshold (PPT) over the two regions (index knee and non-dominant wrist) in random order. The mechanical temporal summation (MTS) procedure will be assessed twice using a nylon monofilament (Semmes monofilament 6.65, 300 g) under two contexts: at rest and during EEG recordings. Ten repetitive contacts will be delivered at 1 Hz. The participants rate the pain severity on an 11-point NPRS (0 = no pain to 100 = extreme pain) immediately after the first contact and rate the greatest pain intensity they experienced during 10 contacts. MTS is the difference between the NPRS scores after the first contact and the greatest pain scores. The average of two trials will be calculated. MTS will also be administered while EEG is being recorded. Pain intensity (NPRS 0–100) will be recorded following a single contact, and then the MTS will be administered in the following order: three trials of 10 contacts with 20 s rest period between trials. The overall pain intensity (NPRS 0–100) experienced during the three trials will be recorded following the completion of EEG recordings.

The conditioned pain modulation (CPM) procedure will be administered according to the published recommendations (98, 115) to measure the efficiency of the endogenous pain modulatory system. The conditioning stimulus involves the participants submerging their dominant hand in a cold water (−5°) bath for 2 min (maximum period) or until it is too uncomfortable. Pain intensity during the conditioning stimulus will be recorded on a 11-point NPRS (0 = no pain to 100 = extreme pain) at every 15 s intervals (98). The test stimulus involves measuring the suprathreshold PPT (pain40) at the tibialis anterior muscle over the non-dominant leg region. Two PPT (pain40) trials prior to exposure to the conditioning stimulus and three trials of PPT (pain40) trials at 30, 60, and 90 s following the conditioning stimulus will be recorded. The CPM will be determined by calculating the percent change score for each time point. Heart rate and blood pressure will be monitored for safety reasons.

For the collection, processing, and analysis of EEG data (125–127), the SynAmps RT Amplifier (Compudemics Neuroscan) will be used to capture resting-state EEG data for 10 min while the participant is seated in an upright position in a quiet room with closed eyes. The raw EEG signals will be processed according to our previous work (125–127). Exact low-resolution brain electromagnetic tomography (eLORETA) software will be used to estimate the intracerebral electrical sources in the following frequency bands: infraslow (0.01–0.10 Hz), slow (0.2–1.5 Hz), delta (2–3.5 Hz), theta (4–7.5 Hz), alpha1 (8–10 Hz), alpha2 (10.5–12 Hz), beta1 (12.5–18 Hz), beta2 (18.5–21 Hz), beta3 (21.5–30 Hz), and gamma (30.5–44 Hz). The current source density (CSD) and functional connectivity (FC) will be calculated for the selected regions of interest (ROIs), namely, pregenual, sub-genual, prefrontal, cingulate, insular, and somatosensory cortices. MM and NF interventions have been studied previously and have demonstrated altering the activity of the ROIs chosen in this trial. The changes in the CSD of the selected ROIs and FC between ROIs will be analysed within and between groups.

## Quantitative data analyses plan

Descriptive statistics will be derived for primary and secondary outcome measures. The sample size estimation was not determined for primary outcomes since it is a feasibility trial. However, we aim to recruit a sample (20 participants per group, 60 in total) in 1 year in a single centre to provide sufficient data for assessing adverse events, feasibility outcomes, identifying operational issues, and estimating variability estimates of clinical pain outcomes.

**In the full clinical trial**, linear mixed models will be used to examine the differences in changes in the pain and physical function between groups to determine the effectiveness of interventions against usual care based on the randomised group (i.e., using an intention-to-treat approach). Group–time interactions will be used to assess between-group differences at 1 and 3 months following the intervention, with 3 months being the primary endpoint. Potential explanatory factors (which could include age, sex, BMI, duration, baseline pain severity, and depression scores) will be adjusted in the analysis. In this feasibility study, we will still perform these analyses (with no stratification or competing exposure variables included) to assess the study protocol, focusing on the uncertainty in estimated effects, not statistical significance. The following parameters will be used to determine the sample size in the full clinical trial: standard deviations (SD) and correlations between repeated measures of outcome measures derived from the feasibility trial. The full trial will have 80% power to detect a meaningful difference in the primary outcome (i.e., pain severity at 3 months). The minimal clinically important difference for pain severity (NPRS) is 1.8 using a two-sided (0.05) significance level. An attrition rate informed by the results of this feasibility study will be incorporated.

Qualitative data analysis (72): The interviews will be audio-recorded and fully transcribed. The General Inductive Approach will guide the analysis. The qualitative findings will provide important information regarding recruitment and retention in the trial and the acceptability of interventions. The results of the qualitative study will be published separately.

Māori analysis (70, 128–131): The journal and the interview data will be subjected to qualitative and inductive thematic analysis and Māori analysis, field notes collected by the Māori researcher, and interviews with Māori participants to ensure that the study reflects the values, beliefs, and cultural practices of the Tangata Whenua community towards broader objectives for Māori development. This will support the researcher in identifying factors related to the perceptions of the Māori participants, including the study site and setting, the overall value of the study, and the level of acceptability of interventions as potential clinical treatments for managing pain associated with knee OA. Overall, the interviews will provide deeper insights into whether assessment and training practices could be acceptable to a Māori worldview.

### Criteria for termination of the study

If any unexpected serious adverse medical event or other incidents occur, the study or one of the arms of the trial will be discontinued. A serious adverse medical event is defined as any occurrence or effect that is life-threatening, including death, requiring hospitalisation, and significant disability. These medical events are considered very unlikely in this study due to the nature of the interventions.

## Discussion

Self-regulatory interventions can improve health outcomes in people with chronic pain (20, 22). The proposed feasibility clinical trial will provide evidence on the feasibility, safety, and acceptability of self-regulatory interventions such as mindfulness meditation training and electroencephalography neurofeedback training in people with knee OA. It is essential to establish the feasibility of conducting a randomised control trial to investigate the effectiveness of these novel interventions for managing chronic secondary musculoskeletal pain. Therefore, a feasibility trial has been designed to assess the factors that could influence the conduct of a fully powered clinical trial.

The recruitment rate is one of the primary outcomes of this feasibility trial. Based on our previous experience in EEG-NF studies (56, 64, 65), we anticipate a favourable response from the community of people with knee OA living in an urban centre in this trial. However, predicting the recruitment rate/enrolment in this trial is not straightforward. Several factors can contribute to a less-than-optimal recruitment rate in this trial. Some of these factors that may affect recruitment include the presence of concurrent trials recruiting people with knee OA; the biomedical beliefs (wear and tear) in people with knee OA (135) could preclude participation in a trial focused on mind–body interventions such as mindfulness meditation; the potential of planned study advertisements and community engagements in reaching the population of interest, including the Māori and Pacific populations, and the anticipated level of acceptability and perceived usefulness of these self-regulatory brain-based interventions by the community of people with knee OA (64) are also important considerations. Moreover, this trial involves a significant time commitment over a short period, which may be a barrier to participation, affecting recruitment rates. Moreover, other barriers, such as parking availability and time away from work/family/childcare, can influence participation rates (133, 134). As hypothesised, good compliance and retention rates (80%) in the active training groups are expected.

Improving the treatment of knee osteoarthritis and access to treatments will help reduce health inequity between the Māori and non-Māori population (128). A Māori investigator will apply a Māori-centric approach to research, drawing on Kaupapa Māori theory (130). The proposed clinical trial will foster whakawhanaungatanga (building relationships) with Māori stakeholders and incorporate practices aligned with a Māori worldview, ensuring culturally relevant and sensitive interventions. Qualitative interview data will guide researchers in developing and testing culturally sensitive interventions in a future clinical trial (95), including addressing the barriers to participating in a clinical trial, thus addressing the specific needs of the Māori community in the New Zealand context. There is still a risk of inadequate representation of Māori in this trial despite this clinical trial being designed to implement culturally appropriate study procedures.

Since it is a feasibility clinical trial (137), and the sample size for this trial was not estimated, the secondary clinical outcomes were not powered to detect treatment effects. However, as supported by previous literature (64, 65), positive trends in the secondary outcomes, including pain severity, interference, and physical function, are anticipated in people undergoing active training. We hypothesise that the up-training pgACC training protocol will increase the current density across the frequency spectrum in the pregenual anterior cingulate cortex in people undergoing neurofeedback training (56). We hypothesise a reduction in the somatosensory cortex activity following mindfulness meditation training, whereas greater activation at the rostral anterior cingulate, anterior insula, and orbitofrontal cortices is expected. We also hypothesise a reduction in the mechanical temporal summation pain scores, an increased pressure pain threshold, and positive conditioned pain modulation responses in the active training groups compared with those in the control group. The dosage of interventions tested in this trial was similar to that of previous trials, but it may not be enough to demonstrate long-term clinical outcomes (64, 65). A future fully powered clinical trial could consider having booster training sessions to assess medium- to long-term training effects on clinical outcomes.

Estimating a range of side effects (minor to adverse events) associated with exposure to the proposed self-regulatory training procedures is critical. We will record symptoms weekly using the Discontinuation-Emergent Sign and Symptom checklist (93). Based on the previous pilot trial, we observed no significant adverse events associated with NF training (64, 65). The brief mindfulness meditation protocol used in this trial has been thoroughly investigated in experimental acute pain conditions and people with pain. This is the first trial that will provide signs and symptoms that may be associated with this specific brief mindfulness meditation training protocol used in this trial (136).

The experienced research team trained the research assistants in administering the assessment procedures. The research team has trained the research assistant to provide training and help in troubleshooting as it arises. The trained RAs will offer training as reported in the TIDieR checklist. An expert investigator will assess the fidelity of the NF intervention administration (138). A clinical psychologist associated with the Centre for Mindfulness, University of Southern California trained the research assistant to offer MM to the participants. The MM training protocol is scripted and was adapted slightly for cultural sensitivity by the Māori investigator; hence, the fidelity assessment of delivering MM may not be essential.

The biopsychosocial outcome measures in this trial have been selected based on the recommended core outcomes for clinical trials of chronic pain (97, 98). In addition, a range of physiological measures were used alongside the clinical outcomes to explore the potential mediating mechanisms contributing to the effect of intervention (120). A future fully powered clinical trial could assess the psychological and physiological markers as mediators and moderators of treatment effects. Due to a range of outcome measures utilised in the trial, we anticipate that the participants will face a burden in terms of completing the questionnaires and undergo test procedures. However, this issue could be addressed by scheduling for some questionnaires to be completed at home and administering specific questionnaires in person (134).

The neurofeedback training protocol in this trial has been previously used and demonstrated a clinically meaningful descriptive trend in the clinical outcomes (65). High-quality evidence (55) suggests that EEG-NF training effectively induces clinically meaningful analgesia in people with chronic pain. The observed clinical effect following NF training could result from active ingredients, such as NF-induced alterations in brain area activity and the placebo effects associated with NF training (e.g., wearing a cap or listening to auditory feedback). Therefore, having a placebo group (wearing an EEG cap only) is not required as this is a feasibility trial assessing the clinical effectiveness of NF training against mindfulness meditation and usual care. In our trial, we believe that the active ingredient (change in the brain activity) induced by the EEG-NF training protocol and the placebo effects would collectively produce the treatment effects.

## Data Availability

The original contributions presented in the study are included in the article/Supplementary Material, further inquiries can be directed to the corresponding author.
